# A phase 2 randomized, double-blind study of AMG 108, a fully human monoclonal antibody to IL-1R, in patients with rheumatoid arthritis

**DOI:** 10.1186/ar3163

**Published:** 2010-10-15

**Authors:** Mario H Cardiel, Paul P Tak, William Bensen, Francis X Burch, Sarka Forejtova, Janusz E Badurski, Tarundeep Kakkar, Terry Bevirt, Liyun Ni, Ellen McCroskery, Angelika Jahreis, Debra J Zack

**Affiliations:** 1 Centro de Investigacion Clinica de Morelia, Morelia, Virrey de Mendoza 1998-Int. 522 Col Félix Ireta, Mich 58070, Mexico; 2 Department of Clinical Immunology/Rheumatology, Academic Medical Center/University of Amsterdam, Meibergdreef 9, Amsterdam 1105 AZ, The Netherlands; 3 St Joseph's Hospital, McMaster University, 25 Charlton Avenue East, Hamilton, ON, L8N 1Y2, Canada; 4 San Antonio Center for Clinical Research, 8527 Village Drive, San Antonio, TX 78217, USA; 5 Revmatologicky ustav, Na Slupi 4, Praha 128 50, Czech Republic; 6 Centrum Osteoporozy i Chorób Kostno-Stawowych, Waryńskiego 6/2, 15-461 Białystok, Poland; 7 Pharmacokinetics and Drug Metabolism, Amgen Inc., One Amgen Center Drive, Thousand Oaks, CA 91320, USA; 8 Global Development Operations, Amgen Inc., One Amgen Center Drive, Thousand Oaks, CA 91320, USA; 9 Global Biostats and Epidemiology, Amgen Inc., One Amgen Center Drive, Thousand Oaks, CA 91320, USA; 10 Global Development, General Medicine and Inflammation Therapeutic Area, Amgen Inc., One Amgen Center Drive, Thousand Oaks, CA, 91320, USA

## Abstract

**Introduction:**

Preclinical work has suggested that IL-1 plays a critical role in the pathogenesis of rheumatoid arthritis (RA). The objective of the present study was to determine the effect of a long-acting IL-1 receptor inhibitor, AMG 108, in a double-blind, placebo-controlled, parallel-dosing study in patients with active RA who were receiving stable methotrexate (15 to 25 mg/week).

**Methods:**

Patients were randomized equally to receive placebo or 50, 125, or 250 mg AMG 108 subcutaneously every 4 weeks for 6 months. The primary efficacy endpoint was a 20% improvement in the American College of Rheumatology response (ACR20) at week 24; other efficacy endpoints included the ACR50, the ACR70, and the RA disease activity score (28-joint count Disease Activity Score) responses, patient-reported outcomes, and pharmacokinetic parameters. Safety endpoints included treatment-emergent adverse events (AEs), infectious AEs, serious AEs, serious infections, injection site reactions, laboratory abnormalities, and antibodies to AMG 108.

**Results:**

Of 813 patients enrolled in the study, 204 patients were randomized to the 50 mg group, 203 to the 125 mg group, 203 to the 250 mg group, and 203 to placebo. At week 24, 40.4% of the 250 mg group, 36% of the 125 mg group, 30.9% of the 50 mg group, and 29.1% of the placebo group achieved an ACR20 (*P *= 0.022, 250 mg vs. placebo). Of the individual ACR components, numerical dose-dependent improvements were only seen in tender joint counts, pain (visual analog scale), and the acute phase reactants, erythrocyte sedimentation rate and C-reactive protein. No dose-related increase was observed in the incidence of treatment-emergent AEs. No deaths were reported, and the incidence of AEs and infections, serious AEs and infections, and withdrawals from study for safety were similar in the AMG 108 and placebo groups.

**Conclusions:**

This large double-blind randomized trial with a long-acting IL-1 receptor blocker, AMG 108, is consistent with the experience of other IL-1 blockers, represents a definitive experiment showing that IL-1 inhibition provides only moderate symptomatic amelioration of arthritis activity in the majority of RA patients, and provides an answer to a question that has been discussed for many years in the rheumatologic community.

**Trial Registration:**

ClinicalTrials.gov NCT00293826

## Introduction

Rheumatoid arthritis (RA) is a chronic, systemic, autoimmune, inflammatory arthropathy of unknown etiology, characterized by progressive destruction of the affected joints, deformity, disability, and premature death [1]. Genetic and environmental factors have been implicated in the pathogenesis of RA [2]. The inflammatory response in the synovial membrane includes hyperplasia, increased vascularity, and infiltration of inflammatory cells [3]. Various inflammatory cascades ultimately lead to activation of macrophages and fibroblast-like synoviocytes to overproduce proinflammatory cytokines such as IL-1, IL-6, and TNFα [4,5]. Other cytokines, as well as matrix metalloproteinases, are produced that are responsible for cartilage degradation and bone erosion.

IL-1 is considered a pivotal cytokine in chronic destructive arthritis; it is a strong activator of chondrocytes, induces cartilage breakdown through upregulation of metalloproteinases, and causes profound suppression of cartilage matrix synthesis. IL-1 is also able to increase receptor activator of NF-κB ligand expression and thus drive osteoclast formation and activation [6,7], leading to bony erosions. Several murine models have shown the arthritogenic and erosive potency of IL-1. In collagen-induced arthritis, a frequently used animal model for RA, TNF was an important contributor to inflammation at the onset of disease, but IL-1 receptor (IL-1R) blockage was highly efficacious in reducing inflammation, both in acute and advanced stages [8]. In antigen-induced arthritis, cartilage damage, erosion progression, and propagation of inflammation are dependent on IL-1 [9,10]. In a recent study of immune complex arthritis, IL-1-deficient mice were strongly protected [11]. In a novel transgenic mouse model of adjuvant arthritis, a pure T-cell model, mice deficient in the IL-1R antagonist displayed uncontrolled IL-1 activity and developed spontaneous T-cell-dependent autoimmune arthritis [12]. Overall, the preclinical data strongly support a role for IL-1 in the pathogenesis of synovial inflammation.

In RA patients, however, IL-1 antagonists display relatively modest effects, although they are very effective in the treatment of systemic-onset juvenile idiopathic arthritis, of adult-onset Still's disease, and of several autoinflammatory disorders [13]. The question remains whether these inhibitors were given at doses and intervals that would be able to achieve robust coverage of the IL-1 pathway. We therefore investigated whether use of more continuous blockade of IL-1 could translate into increased efficacy in the treatment of RA.

AMG 108 (Amgen Inc., Thousand Oaks, CA, USA) is a fully human IgG_2 _monoclonal antibody that binds IL-1R type 1 and nonselectively inhibits the activity of both forms of IL-1 (IL-1α and IL-1β). The objective of the present study was to compare the efficacy and safety of three dose levels of AMG 108 with placebo in patients with active RA who were receiving stable methotrexate (MTX) (15 to 25 mg/week).

## Materials and methods

### Patients

Patients were enrolled at 132 study sites in North America (43% of patients; United States, Canada, Mexico), Eastern Europe (43% of patients; Poland, Czech Republic, Hungary, Slovakia, Estonia, Latvia), Western Europe (12% of patients; Netherlands, Spain, Italy, United Kingdom, France, Belgium, Ireland, Sweden), and Australia (2% of patients).

Eligible patients were ≥18 and ≤70 years old and had RA that met the American College of Rheumatology (ACR) classification criteria [14], with active RA for a duration ≥6 months. Active RA was defined as ≥6 swollen joints and ≥6 tender or painful joints and at least one of the following: erythrocyte sedimentation rate (ESR) ≥28 mm/hour, C-reactive protein (CRP) >2.0 mg/dl, or duration of morning stiffness ≥45 minutes at time of screening. Patients must have received MTX for at least 12 consecutive weeks, with a stable dose of oral or subcutaneous MTX at 15 to 25 mg/week for ≥4 weeks at time of screening. Exceptions were granted for a lower dose if it was the highest tolerated dose (toxicity documentation was required). Patients were allowed to be taking stable doses of nonsteroidal anti-inflammatory drugs or oral corticosteroids (≤10 mg prednisone or equivalent) if doses were stable ≥4 weeks before screening.

Patients were excluded from the study if they had received any previous AMG 108 or other commercial or experimental biologic therapies for RA or other inflammatory disease, or had uncontrolled or clinically significant systemic disease other than RA (for example, diabetes mellitus, cardiovascular disease, or hypertension). Patients could not have class IV RA as defined by ACR revised criteria for global functional status in RA [15], Felty's syndrome, a prosthetic joint infection within 5 years or native joint infection within 1 year of screening, or a major chronic inflammatory disease or connective tissue disease other than RA (with the exception of secondary Sjögren's syndrome). Patients could not have: uncontrolled or clinically significant asthma; known sensitivity to mammalian cell-derived drug products; malignancy within 5 years of screening (except for squamous or basal cell carcinoma or successfully treated in situ cervical cancer); serious infection (defined as requiring hospitalization) or recurrent, acute, or chronic infections within 8 weeks of screening; history of *Mycobacterium tuberculosis *or exposure; or known positivity for hepatitis B surface antigen, hepatitis C virus, or human immunodeficiency virus.

Patients were ineligible if they had any of the following clinically significant laboratory values at screening: white blood cell count <3.0 × 10^9^/l, absolute neutrophil count <2.5 × 10^9^/l, platelet count <125 × 10^9^/l, aspartate aminotransferase or alanine aminotransferase >1.5 × upper limit of normal, serum creatinine >1.5 × upper limit of normal, or any other laboratory abnormality that, in the opinion of the investigator, would prevent the patient from completing the study or would interfere with the interpretation of the study results. Patients could not have received intra-articular or systemic corticosteroid injections or any investigational therapy within 4 weeks of screening, any disease-modifying antirheumatic drug other than MTX within 6 weeks of screening, cyclophosphamide within 6 months of screening, or any live vaccine within 3 months of the first dose of investigational product. Patients were ineligible if, in the investigator's opinion, they had any physical or psychiatric disorder that could interfere with their ability to give informed consent or participate in the study. Patients with active substance abuse (within 6 months of screening) or any condition that might require narcotic analgesics were excluded. Pregnant or nursing women were not eligible, and all sexually active patients were required to use adequate contraception.

### Study design

The investigation was a double-blind, placebo-controlled, parallel-dosing study in patients with active RA who were receiving stable doses of MTX (15 to 25 mg/week), were biologic-naïve, and had discontinued disease-modifying antirheumatic drugs other than MTX prior to study entry. Patients were randomized equally to receive placebo or 50, 125, or 250 mg AMG 108 subcutaneously every 4 weeks for 6 months.

The study was conducted according to the Declaration of Helsinki and the International Conference on Harmonisation Tripartite Guideline on Good Clinical Practice [16]. Approvals from appropriate research ethics committees were obtained from each participating study center (Additional file 1). All patients provided written informed consent before participating. An external Data Monitoring Committee monitored patient safety throughout the duration of the study.

### Endpoints

The primary efficacy endpoint was the ACR20 response [17] at week 24. Secondary efficacy endpoints included ACR50 and ACR70 responses and the individual components of the ACR; the Disease Activity Score using the 28-joint count (DAS28) (using CRP primarily; ESR was used only if CRP was missing) [18]; and patient-reported outcomes, including the Disability Index of the Health Assessment Questionnaire [19], and the physical and mental composite scores of the Short Form-36 [20] at week 24. All of these endpoints over time in the study were analyzed as exploratory endpoints.

Safety endpoints included treatment-emergent adverse events (AEs), infectious AEs, serious AEs, serious infections, injection site reactions, laboratory abnormalities, and anti-AMG 108 antibodies.

The pharmacokinetic profile of AMG 108 in combination with MTX was also assessed (in 520 patients with sparse sampling, and in 37 patients with intensive sampling). A validated ELISA was used to quantify AMG 108 in the serum; IL-1R was used as the capture receptor, and biotinylated IL-1R was used for detection.

Binding antibodies to AMG 108 (anti-drug antibodies) were evaluated using a validated acid dissociation electrochemiluminescence-based bridging immunoassay. Serum samples were collected predose and at weeks 12, 24, and 34. A cell-based bioassay was used to detect neutralizing antibodies in samples that were positive in the immunoassay.

### Sample size

The primary endpoint was the ACR20 response at week 24. The sample size of 196 patients per treatment arm was calculated to provide at least 80% power at a statistical significance level of 5% (two-sided) to test whether monthly subcutaneous dosing of AMG 108 in combination with MTX demonstrated an ACR20 response at week 24 that was >20% above that with MTX therapy alone in RA subjects. The sample size was inflated to allow for a 10% dropout rate over the course of the study.

### Statistical analysis

Patients were analyzed according to the randomized treatment arm regardless of actual treatment received during the study. All efficacy endpoints were analyzed using the intent-to-treat analysis set, which included all randomized patients regardless of whether they received investigational product. The safety dataset included all patients who received ≥1 dose of investigational product.

The primary efficacy endpoint compared the ACR20 response rate at week 24 in the 250 mg AMG 108 group with that of the placebo group. All secondary endpoints were tested sequentially in a prespecified order to control the overall family-wise type 1 error rate at 5% (two-sided). The comparisons of proportions (for dichotomous variables) among treatment arms were carried out using Fisher's exact test. The comparisons of distribution-location parameters (for continuous and ordinal variables) among treatment arms were performed using the Wilcoxon rank-sum text. For dichotomous variables, missing values were imputed using a nonresponder imputation method; for continuous variables, the primary analysis is based on observed cases.

## Results

### Patient disposition and disease characteristics

**Figure 1:**
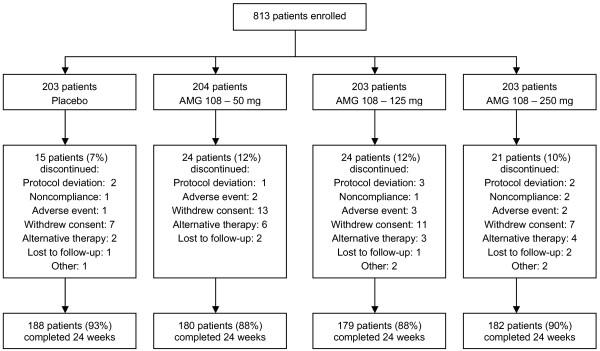
**Patient disposition**. CONSORT diagram.

The patient disposition is presented in Figure 1. Randomization was well balanced across groups: 204 patients were randomized to 50 mg AMG 108, 203 patients to 125 mg AMG 108, 203 patients to 250 mg AMG 108, and 203 patients to placebo. Of 813 patients enrolled in the study, 805 (99%) received ≥1 dose of investigational product. Study completion at week 24 was similar across treatment groups: 88 to 90% in the AMG 108 groups vs. 93% in the placebo group.

Demographics and baseline disease characteristics are presented in Table 1. Most patients were women (≥76% in each group), and most were white (≥83% in each group). The mean age was 51.8 years in the AMG 108 groups and was 52.1 years in the placebo group.

**Table 1:** Baseline demographics and disease characteristics

		AMG 108			
		
	Placebo (*n *= 203)	50 mg (*n *= 204)	125 mg (*n *= 203)	250 mg (*n *= 203)	Total (*n *= 610)
Mean age (years)	52.1	51.4	51.7	52.2	51.8
Age group, *n *(%)					
< 65 years	185 (91.1)	171 (83.8)	176 (86.7)	184 (90.6)	531 (87.0)
≥65 years	18 (8.9)	33 (16.2)	27 (13.3)	19 (9.4)	79 (13.0)
Female, *n *(%)	158 (77.8)	155 (76.0)	163 (80.3)	160 (78.8)	478 (78.4)
Ethnicity, *n *(%)					
White	168 (82.8)	169 (82.8)	177 (87.2)	179 (88.2)	525 (86.1)
Hispanic	27 (13.3)	24 (11.8)	18 (8.9)	17 (8.4)	59 (9.7)
Other^a^	8 (3.9)	11 (5.4)	8 (3.9)	7 (3.4)	26 (4.3)
Mean weight (kg)	76.4	77.2	74.2	75.0	75.4
Mean height (cm)	164.0	164.6	164.0	164.8	164.5
Mean body mass index (kg/m^2^)	28.4	28.4	27.5	27.5	27.8
Mean duration of RA (years)	7.6	7.3	7.5	8.0	7.6
Subcomponent of ACR (mean)					
Tender joint count	26.3	26.5	24.8	26.9	26.1
Swollen joint count	16.8	16.8	15.8	15.7	16.1
Patient global assessment	5.9	6.2	6.1	6.1	6.2
Physician global assessment	6.2	6.3	6.3	6.5	6.4
Patient pain assessment	51.2	53.9	53.5	57.0	54.8
HAQ Disability Index	1.4	1.5	1.5	1.5	1.5
C-reactive protein (mg/dl)	1.2	1.8	1.5	1.4	1.6
ESR (mm/hour)	33.4	40.1	35.6	35.5	37.1
Mean DAS28 CRP	4.7	4.9	4.7	4.8	4.8
Mean DAS28 ESR	5.3	5.4	5.3	5.4	5.3
Mean tender joint count (28 joints)	14.0	14.5	13.5	14.0	14.0
Mean swollen joint count (28 joints)	11.3	10.6	10.8	11.2	11.0

ACR, American College of Rheumatology; DAS28, Disease Activity Score (28-joint count); ESR, erythrocyte sedimentation rate; HAQ, Health Assessment Questionnaire; RA, rheumatoid arthritis. ^a^Black, Asian, American Indian or Alaska native, native Hawaiian or other Pacific Islander.

### Efficacy

In the primary efficacy analysis at week 24 (using the nonresponder imputation method), the ACR20 response rate was statistically significantly higher in the 250 mg AMG 108 group (40.4%) compared with the placebo group (29.1%; *P *= 0.022). Small improvements in the ACR20 response at week 24 were also seen in the 125 mg AMG 108 group (36.0%; Table 2). The ACR20, ACR50, and ACR70 responses are presented in Table 2. Only the 250 mg AMG 108 group showed a significant improvement in ACR50 scores, and no groups were different from placebo with respect to ACR 70.

**Table 2:** ACR responses, DAS28 C-reactive protein, and EULAR28 responses at week 24

		AMG 108		
		
	Placebo (*n *= 203)	50 mg (*n *= 204)	125 mg (*n *= 203)	250 mg (*n *= 203)
ACR response, *n *(%)^a^				
ACR20	59 (29.1)	63 (30.9) (*P *= 0.746)	73 (36.0) (*P *= 0.168)	82 (40.4) (*P *= 0.022)
ACR50	17 (8.4)	24 (11.8) (*P *= 0.323)	28 (13.8) (*P *= 0.113)	41 (20.2) (*P *< 0.001)
ACR70	8 (3.9)	4 (2.0) (*P *= 0.259)	5 (2.5) (*P *= 0.575)	12 (5.9) (*P *= 0.492)
DAS28 CRP, mean change from baseline^b^	-0.60	-0.69 (*P *= 0.213)	-0.92 (*P *< 0.001)	-1.18 (*P *< 0.001)
EULAR28 response, *n *(%)^c^				
Good	16 (8%)	20 (10%)	28 (13.9%)	39 (19.5%)
Moderate	61 (30.7%)	59 (29.3%)	76 (37.8%)	76 (38%)
No response	122 (61.3%)	122 (60.7%)	97 (48.3%)	85 (42.5%)

ACR, American College of Rheumatology; CRP, C-reactive protein; DAS28, Disease Activity Score (28-joint count); EULAR28, European League Against Rheumatism (28-joint count). ^a^Nonresponder imputation: missing ACR responses were imputed as nonresponder. *P *values are nominal without multiplicity adjustment, using Fisher's exact test. ^b^Observed cases. *P *values are comparisons with placebo, using two-sided Wilcoxon rank-sum test. ^c^EULAR28 response is classified as good, moderate, and no response based on the DAS28 ESR at week 24 and the DAS28 ESR improvement from baseline at week 24 [31].

**Figure 2:**
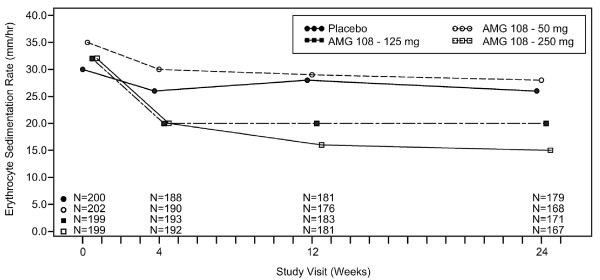
**Median erythrocyte sedimentation rate over time**.

Additionally, numerical dose-dependent responses were observed at week 24 in several individual components of the ACR, including tender joint count, pain (visual analog scale), ESR, and CRP; all components of the ACR are presented in Table 3. Responses were weak or absent in swollen joint count and physician and patient global assessments. Median ESR values are shown over time in Figure 2.

**Table 3:** Improvements in American College of Rheumatology and DAS28 components at week 24

		AMG 108		
		
	Placebo (*n *= 203)	50 mg (*n *= 204)	125 mg (*n *= 203)	250 mg (*n *= 203)
Tender joint count (68 joints)				
Mean (SD) % change	-20.0 (127.5)	-29.5 (60.4)	-35.7 (43.6)	-40.2 (44.3)
Median % change	-31.3	-38.4	-40.0	-46.7
Swollen joint count (66 joints)				
Mean (SD) % change	-32.2 (50.0)	-33.7 (46.5)	-33.8 (114.8)	-39.1 (53.3)
Median % change	-33.1	-40.8	-46.3	-47.1
Patient global assessment				
Mean (SD) % change	-11.6 (49.8)	-16.7 (43.8)	-21.6 (48.2)	-17.8 (64.5)
Median % change	-20.0	-16.7	-28.6	-20.0
Physician global assessment				
Mean (SD) % change	-30.1 (33.7)	-36.0 (30.4)	-36.2 (33.2)	-43.0 (30.8)
Median % change	-33.3	-37.5	-40.0	-50.0
Pain (visual analog scale)				
Mean (SD) % change	2.2 (142.6)	-11.4 (76.3)	-20.6 (60.6)	-30.9 (46.9)
Median % change	-12.8	-20.0	-28.9	-34.7
HAQ Disability Index				
Mean (SD) % change	-11.1 (53.0)	-12.2 (40.6)	-20.5 (48.2)	-25.2 (44.6)
Median % change	-11.4	-13.3	-20.0	-25.0
Erythrocyte sedimentation rate				
Mean (SD) % change	12.7 (77.7)	5 (124.3)	-18.3 (68.7)	-30.4 (73.7)
Median % change	-6.3	-13.2	-36.8	-46.7
C-reactive protein				
Mean (SD) % change	123.2 (695.3)	32.2 (164.0)	-1.9 (181.5)	-25.5 (168.5)
Median % change	5.1	-12.4	-41.2	-59.4
Tender joint count (28 joints)				
Mean (SD) % change	-13.2 (143.7)	-21.0 (73.2)	-31.6 (58.7)	-36.5 (65.7)
Median % change	-33.3	-33.3	-40.0	-44.4
Swollen joint count (28 joints)				
Mean (SD) % change	-28.2 (52.7)	-30.0 (48.1)	-28.7 (118.3)	-38.0 (52.2)
Median % change	-33.3	-33.3	-44.4	-44.4

DAS28, Disease Activity Score (28-joint count); HAQ, health assessment questionnaire; SD, standard deviation.

Results of the DAS28 CRP at week 24 are presented in Table 2. The 125 mg and 250 mg AMG 108 groups had significantly greater mean improvements from baseline compared with placebo at week 24 (-0.92 and -1.18, respectively, vs. -0.60; *P *< 0.001). Of note, most patients had moderate disease activity at study entry, as shown by their baseline DAS28 CRP scores (Table 1). The European League Against Rheumatism 28-joint count responses (EULAR28) are also presented in Table 2.

Clinically meaningful improvements were observed in some patient-reported outcome measures. Mean improvements from baseline at week 24 in the Health Assessment Questionnaire Disability Index were greater in the 250 mg and 125 mg AMG 108 groups (-0.40 and -0.34, respectively; *P *< 0.001, each group compared with placebo) than in the 50 mg group (-0.24) or the placebo group (-0.19). Mean improvements from baseline at week 24 in the physical composite score of the Short Form-36 were numerically greater in all AMG 108 groups (range 4.6 to 7.2) than in the placebo group (3.3), and were significantly greater in the 250 mg group (7.2) compared with placebo (*P *< 0.001). Improvements in the mental composite score, however, were similar among the AMG 108 and placebo groups (data not shown).

### Pharmacokinetics

Following single-dose and multiple-dose administration, AMG 108 is slowly absorbed - with the median time at which the maximum concentration occurs ranging from 3.8 to 3.9 days (50 mg group), from 3.9 to 4.0 days (125 mg group), and from 5.9 to 7.0 days (250 mg group). Trough pharmacokinetic levels in the 250 mg dose group were approximately 10-fold above the predicted 90% inhibitory concentration for IL-1 (Table 4). Importantly, trough pharmacokinetic levels were maintained in the 125 mg and 250 mg groups, suggesting prolonged coverage of the IL-1 pathway.

**Table 4:** Summary of AMG 108 trough concentration (nM) at 20 and 24 weeks

	50 mg AMG 108		125 mg AMG 108		250 mg AMG 108	
	
	Week 20 (*n *= 144)	Week 24 (*n *= 137)	Week 20 (*n *= 146)	Week 24 (*n *= 140)	Week 20 (*n *= 137)	Week 24 (*n *= 138)
Mean	0	0	16.5	19.8	155	160
Median	0	0	6.25	4.65	139	141
Range	0 to 6.43	0 to 0.32	0 to 104	0 to 268	0 to 417	0 to 1,000

Below quantifiable levels reported as 0. 90% inhibitory concentration = 13.5 nM, 50% inhibitory concentration = 1.5 nM.

### Safety

AMG 108 was well tolerated at all doses administered during the study. No increase in incidence of treatment-emergent AEs was observed with increasing AMG 108 dose (Table 5). No deaths were reported, and the incidence of AEs, infectious AEs, serious AEs and infections, and withdrawals from study due to AEs were no higher in AMG 108 groups than in the placebo group (Table 5).

**Table 5:** Summary of adverse events through 24 weeks

		AMG 108			
		
	Placebo (*n *= 201)	50 mg (*n *= 202)	125 mg (*n *= 201)	250 mg (*n *= 201)	Total^a^ (*n *= 604)
Any AE	133 (66.2)	134 (66.3)	143 (71.1)	135 (67.2)	412 (68.2)
Most common AE					
Headache	16 (8.0)	11 (5.4)	15 (7.5)	15 (7.5)	41 (6.8)
Diarrhea	13 (6.5)	10 (5.0)	11 (5.5)	15 (7.5)	36 (6.0)
Nasopharyngitis	18 (9.0)	11 (5.4)	13 (6.5)	12 (6.0)	36 (6.0)
URI	15 (7.5)	10 (5.0)	13 (6.5)	13 (6.5)	36 (6.0)
Treatment-related AE	48 (23.9)	43 (21.3)	50 (24.9)	45 (22.4)	138 (22.8)
AE leading to:					
Study discontinuation	1 (0.5)	3 (1.5)	3 (1.5)	3 (1.5)	9 (1.5)
Withdrawal of study drug	2 (1.0)	4 (2.0)	4 (2.0)	3 (1.5)	11 (1.8)
Hospitalization	9 (4.5)	5 (2.5)	5 (2.5)	6 (3.0)	16 (2.6)
Any infection	72 (35.8)	64 (31.7)	68 (33.8)	62 (30.8)	194 (32.1)
Infection leading to:					
Study discontinuation	1 (0.5)	0	0	1 (0.5)	1 (0.2)
Withdrawal of study drug	0	1 (0.5)	0	1 (0.5)	2 (0.3)
Hospitalization	3 (1.5)	1 (0.5)	1 (0.5)	2 (1.0)	4 (0.7)
Injection site reaction	5 (2.5)	8 (4.0)	10 (5.0)	9 (4.5)	27 (4.5)
Any serious AE	11 (5.5)	6 (3.0)	6 (3.0)	9 (4.5)	21 (3.5)
Treatment-related serious AE	2 (1.0)	2 (1.0)	1 (0.5)	1 (0.5)	4 (0.7)
Serious infection	3 (1.5)	2 (1.0)	1 (0.5)	2 (1.0)	5 (0.8)
Death	0	0	0	0	0

Data presented as *n *(%). AE, adverse event; URI, upper respiratory infection. ^a^All randomized patients who received at least one dose of AMG 108.

Injection-site reactions occurred more frequently in AMG 108 groups than in the placebo group (Table 5), but most reactions were mild or moderate in severity, with the majority of cases lasting <5 days. Of the 604 patients who received ≥1 dose of AMG 108, 83 patients (13.7%) were positive for binding antibodies to AMG 108 at some time during the study; 22 patients (4%) were positive for neutralizing antibodies (data not shown).

**Figure 3:**
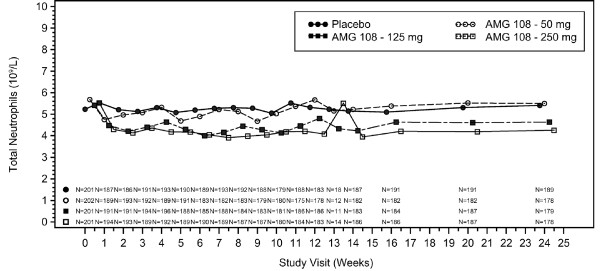
**Median neutrophil counts over time by visit and by treatment group**.

No clinically significant changes in laboratory abnormalities were observed, with the exception of expected decreases in the absolute neutrophil count and platelet counts that were dose related; these decreases recovered to baseline values by the end-of-study evaluation (week 34; 10 weeks following the last dose of AMG 108). The median decreases in neutrophil counts over time are shown by visit and by treatment group in Figure 3.

## Discussion

In the present study of AMG 108 - a long-acting IL-1R inhibitor - improvements in the signs and symptoms of RA as measured by the ACR20 were greater in the 250 mg AMG 108 group (40.4%) at week 24, compared with the placebo group (29.1%; *P *= 0.022). Improvements in the ACR50 were also statistically significant for the 250 mg AMG 108 group compared with placebo; however, the numbers of patients with ACR50 responses were low (20.2% vs. 8.4%, 250 mg AMG 108 vs. placebo, respectively; *P *< 0.001). The ACR70 response was not significantly different between the two groups. Of note, mean responses on the Health Assessment Questionnaire Disability Index observed in all groups treated with AMG 108 were above the minimum clinically important difference (0.22) published for this outcome measure [21]. It is interesting that the components of the ACR responses showing dose-dependent effects in this study were those that are consistent with the known functions of IL-1 in pain [22] and in the acute phase response [23]: AMG 108 decreased pain, tender joints, ESR, and CRP. The other components of the ACR scoring system were not affected. AMG 108 was well tolerated in this patient population, with a safety profile similar to that of placebo. No dose-related increases were observed in the incidence of AEs, and no unanticipated events were reported.

Of interest, the effectiveness of IL-1R blockade with a long-acting receptor blocker, AMG 108, appears to be moderate and similar to those described with other IL-1 blockers (such as anakinra, pralnacasan, and IL-1 TRAP) [24-26], despite a constant concentration of drug estimated to be 10-fold higher than the predicted 90% inhibitory concentration for IL-1. The limited efficacy of IL-1 blockers in RA therefore appears not to be explained solely by pharmacokinetic or pathway coverage, since the efficacy provided by AMG 108 was similar to that in other IL-1 inhibitor studies [27] despite constant inhibition of the IL-1 pathway for a 6-month period with AMG 108.

The results of treatment with AMG 108 instead appear to point to a limited role of IL-1 in human RA synovial inflammation - a notion supported by the profound effect seen with other IL-1 inhibitors in conditions other than RA, such as systemic-onset juvenile idiopathic arthritis, gout, neonatal-onset multisystem inflammatory disease, cryopyrin-associated periodic syndromes, and other autoinflammatory disorders [28,29]. Although it is not known how many IL-1Rs have to be blocked to prevent IL-1 binding and signaling in a substantial way, AMG 108 achieved steady concentrations in the 250 mg/month dosing arm that should have been 10-fold higher than those needed to inhibit 90% of IL-1 signaling. In addition, dose-dependent changes were seen in those parameters known to be affected by IL-1, such as pain and acute phase reactants. This discrepancy of the IL-1 effect between the animal models and RA patients may therefore be due to differences in timing of treatment, with animals treated at very early stages of disease and RA patients at later stages of chronic synovial inflammation, or may instead be due to differences between the cytokine interactions in rodents versus humans [30].

A limitation of the present study is the absence of radiographic evaluation. The primary analysis was focused on clinical signs and symptoms rather than on joint damage measured radiographically; we therefore do not know whether a more robust benefit than shown in the clinical findings would have been achieved if radiographs had been evaluated. Our results may possibly reflect a partial uncoupling of clinical and radiographic findings.

## Conclusions

The present large, double-blind, randomized trial with a long-acting IL-1R blocker, AMG 108, is consistent with the experience of other IL-1 blockers, represents a definitive experiment showing that IL-1 inhibition provides only moderate symptomatic amelioration of arthritis activity in the majority of RA patients, and provides an answer to a question that has been discussed for many years in the rheumatologic community - whether use of more continuous blockade of IL-1 could translate into increased efficacy in the treatment of RA.

## Abbreviations

ACR: American College of Rheumatology; AE: adverse event; CRP: C-reactive protein; DAS28: Disease Activity Score (28-joint count); ELISA: enzyme-linked immunosorbent assay; ESR: erythrocyte sedimentation rate; IL: interleukin; IL-1R: interleukin-1 receptor; MTX: methotrexate; NF: nuclear factor; RA: rheumatoid arthritis; TNF: tumor necrosis factor.

## Competing interests

MHC, PPT, WB, FXB, SF and JEB received research funding from Amgen Inc; in addition, MHC, PPT and WB have received consultant or speaker fees from Amgen Inc. TK, TB, LN, EM, AJ and DJZ are current or former full-time employees of, and hold stock in, Amgen Inc. Amgen Inc. is a patent-holder on AMG 108.

## Authors' contributions

TK, TB, LN, EM, AJ and DJZ made substantial contributions to the study concept or design. MHC, PPT, WB, FXB, SF and JEB assisted with the acquisition of the data. TK and LN performed data analysis. All authors assisted with interpretation of the data, helped to draft and revise the manuscript for intellectual content. All authors read and approved the final manuscript.

## Supplementary Material

Additional file 1 **Investigator site list**. Table listing the principal investigators and full study center details. Click here for file
