# Supplementary material for: A phase 2 randomized, double-blind study of AMG 108, a fully human monoclonal antibody to IL-1R, in patients with rheumatoid arthritis
Source: Arthritis Res Ther. 2010 Oct 15;12(5):R192. doi: 10.1186/ar3163 (PMC2991028; doi:10.1186/ar3163)
Supplement: Additional file 1 — Investigator site list. Table listing the principal investigators and full study center details. [file ar3163-S1.DOC]

**Cardiel et al: A phase 2 randomized, double-blind study of AMG 108, a fully human monoclonal antibody to IL‑1R, in patients with rheumatoid arthritis**

**Investigator Site List**

|  | |  | **Study Site** | | |
| --- | --- | --- | --- | --- | --- |
| **Principal Investigator** | | | **Name** | **Address** | **Country** |
| Carlos | Abud Mendoza | | Hospital Central "Dr Ignacio Morones Prieto," Unidad Regional de Reumatologia y Osteoporosis | Av V Carranza 2395, colonia Universitaria, San Luis Potosi | 78240 Mexico |
| Silvano | Adami | | Ospedale di Valeggio | Via Santa Maria Crocifissa di Rosa, Valeggio Sul Mincio (VR) | 37067 Italy |
| José Mª | Alvaro-Gracia | | Hospital Universitario de La Princesa | Diego de León, 62., Madrid | 28006 Spain |
| Janusz | Badurski | | Centrum Oseoporozy i Chorob Kostno Stawowych | Warynskiego str 6/2, Bialystok | 15-461 Poland |
| Andre | Beaulieu | | Centre De Rhumatologie St-Louis | 3165 Chemin St-Louis, Ste. 140, Ste Foy, QC | G1W 4R4 Canada |
| William | Bensen | | Charlton Medical Center | 25 Charlton Avenue East, Suite 203, Hamilton, ON | L8N 1Y2 Canada |
| Jacques | Bentin | | CHU Brugmann | place Arthur Van Gehuchten 4, Bruxelles | 1020 Belgium |
| János | Bereczki | | Markhot Ferenc County Hospital, Department of Rheumatology | Fürdö u. 2-4, Eger | H-3300 Hungary |
| Gerolamo | Bianchi | | Ospedale La Colletta ASL 3 Genovese | Via del Giappone, 3, Arenzano (GE) | 16011 Italy |
| Francisco | Blanco | | Complejo Hospitalario Universitario Juan Canalejo | Xubias de Arriba, 84., A Coruña | 15006 Spain |
| Marie-Christophe | Boissier | | Hopital Avicenne | 125 rue de Stalingrad, Bobigny Cedex | 93009 France |
| Pierre | Bourgeois | | Groupe Hospitalier Pitié Salpétrière | 47 boulevard de l'Hôpital, Paris Cedex 13 | 75651 France |
| Barry | Bresnihan | | St. Vincent's University Hospital | Elm Park, Dublin | 4 Ireland |
| Alan | Brodsky | | Arthritis Care and Diagnostic Center | 8440 Walnut Hill Lane, Suite 340, Dallas, TX | 75231 USA |
| Jacques | Brown | | Groupe de recherche en rhumatologie et maladies osseuses | 1200 Avenue de Germain-des-Pres, suite 101, Quebec, QC | G1V 3M7 Canada |
| Gaw | Bruyn | | Medisch Centrum Leeuwarden | Henri Dunantweg 2, Leeuwarden | 8934 AD Netherlands |
| Jan | Brzezicki | | Wojewodzki Szpital Zespolony w Elblagu | ul. Krolewiecka 146, Elblag | 82-300 Poland |
| Marek | Brzosko | | Klinika Chorob Wewnetrznych SPSK Nr 1 AM | ul. Unii Lubelskiej 1, Szczecin | 71-252 Poland |
| Francis | Burch | | San Antonio Center for Clinical Research | 8527 Village Dr., Suite 207, San Antonio, TX | 78217 USA |
| Howard | Busch | | Family Arthritis Center | 1025 Military Trail, Ste. 209,  Jupiter, FL | 33458 USA |
| Juan D. | Cañete | | Hospital Clínic i Provincial de Barcelona | Villarroel, 170, Barcelona | 08036 Spain |
| Mario H | Cardiel | | Hospitales Star Medica | Virrey de Mendoza 2000-502, Felix Ireta, Morelia, Michoacan | 58070 Mexico |
| Mario | Carrabba | | Azienda Ospedaliera Luigi Sacco | Via G.B. Grassi 74, Milano | 20157 Italy |
| John | Carter | | University of South Florida Physicians Group | 12901 Bruce B Downs Blvd. MDC81, Tampa, FL | 33612 USA |
| Melvin | Churchill Jr | | Arthritis Center of Nebraska | 3901 Pine Lake Road, Suite 120, Pine Lake Medical Plaza, Lincoln, NE | 68516 USA |
| Christine | Codding | | Health Research of Oklahoma | 1211 North Shartel Avenue, Ste. 700, Oklahoma City, OK | 73103 USA |
| Steven J | Cohen | | Trinity Clinic Arthritis & Rheumatology | 1327 Troup Highway, Tyler, TX | 75701 USA |
| E M | Colin | | Erasmus MC | Dr Molewaterplein 40 Apotheek, Rotterdam | 3015 GD Netherlands |
| Maurizio | Cutolo | | DIMI, Dipartimento di Medicina Interna e Specialità Mediche, Università degli Studi Genova | Viale Benedetto XV n. 6, Genova | 16132 Italy |
| Eva | Dokoupilova | | Rheumatology | Havlickova 3, Uherske Hradiste | 686 01 Czech Republic |
| William | Edwards | | WW Country Research Center | 2860 Tricom Street, Charleston, SC | 29406 USA |
| Michael | Fairfax | | ArthroCare | 3330 North 2nd Street Suite 601, Phoenix, AZ | 85012 USA |
| Patrice | Fardellone | | Hopital Nord | Place Victor Pauchet, Amiens | 80054 France |
| Justus | Fiechtner | | Fiechtner Justus J, MD | 3394 E Jolly Road, Suite C, Lansing, MI | 48910 USA |
| Anna | Filipowicz-Sosnowska | | Instytut Reumatologii | ul Spartanska 1, Warszawa | 02-637 Poland |
| Diana Elsa | Flores  Alvarado | | Hospital y Clinica OCA SA DE CV | Pablo A Gonzalez 709, Col. Mitras Sur, Monterrey, Nuevo Leon, CP | 64020 Mexico |
| Sarka | Forejtova | | Revmatologicky ustav | Na Slupi 4, Praha | 128 50 Czech Republic |
| Douglas | Freeman, Jr | | NC Arthritis & Allergy Care Center | 3831 Merton Drive, Raleigh, NC | 27609 USA |
| Ignacio | Garcia de la Torre | | Centro de Estudios de Investigacion Basica y Clinica, SC | Justo Sierra 2821-4 Colonia Vallarta Norte, Guadalajara, Jalisco | 44690 Mexico |
| Philippe | Gaudin | | Hôpital Sud | Avenue de Kimberly, Echirolles | 38130 France |
| Norman | Gaylis | | Norman Gaylis, MD | 2845 Aventura Boulevard, Suite 100, Aventura, FL | 33180 USA |
| Emmanuel | George | | Arrowe Park Hospital | Arrowe Park, Upton, Merseyside | L49 5PE United Kingdom |
| Piotr | Gluszko | | Malopolskie Centrum Medyczne s.c. | ul. T. Rejtana 2, Krakow | 30-510 Poland |
| Juan | Gómez-Reino | | Complejo Hosp. Univ. de Santiago de Compostela (Xeral de Galicia) | Travesía de la Choupana, s/n., Santiago de Compostela | 15706 Spain |
| Winfried | Graninger | | Medizinische Universitaet Graz | Auenbruggerplatz 15, Graz | 8036 Austria |
| Eric | Grant | | Campbell Drive Medical Clinic | 115 Campbell Road, Rothesay, NB | Canada |
| Walter | Grassi | | Ospedale Augusto Murri - ASL 5 | Via dei Colli 52 , Jesi AN | 60035 Italy |
| John | Hanly | | Queen Elizabeth Health Sciences Center | 1341 Summer Street, Nova Scotia Rehab Center, Halifax, NS | B3H 4K4 Canada |
| Boulos | Haraoui | | Institut de Rhumatologie de Montreal | 1551 East Ontario Street, 2nd Floor, Montreal, QC | H2L 1S6 Canada |
| Brian | Hazleman | | Addenbrooke's Hospital Cancer Clinical Trials Centre | PO Box 279, Hills Road, Cambridge | CB2 2QQ United Kingdom |
| Per-Johan | Hedin | | Falu Lasarett | -- , Falun | S-791 82 Sweden |
| Joanna | Hensel | | SP ZOZ w Dzialdowie | ul. Lesna 1, Dzialdowo | 13-200 Poland |
| Manfred | Herold | | Medizinische Universitaet Innsbruck, Universitaetsklinik für Innere Medizin | Anichstrasse 35, Innsbruck | 6020 Austria |
| Carol | Hitchon | | Health Sciences Center | RR-149--800 Sherbrook Street, Winnipeg, MB | R3A 1M4 Canada |
| Pawel | Hrycaj | | Akademia Medyczna im. Karola Marcinkowskiego w Poznaniu | ul. Przybyszewskiego 39, Poznan | 60-356 Poland |
| John | Huff | | Arthritis & Osteoporosis Center of South Texas | 14615 San Pedro, Ste. 105, San Antonio, TX | 78232 USA |
| Joseph | Huffstutter | | Arthritis Associates | 1035 Executive Drive, Hixson, TN | 37343 USA |
| Mark | Iannini | | Carondelet Medical Group | 630 N. Alvernon Way, Suite #371,Tucson, AZ | 85711 USA |
| Jolanta | Janik | | Centrum Medyczne OSTEOMED Sp. z o.o. | ul. Bialobrzeska 40A, Warszawa | 00-632 Poland |
| Slawomir | Jeka | | NZOZ 'Nasz Lekarz' Praktyka Grupowa Lekarzy Rodzinnych z Przychodnia Specjalistyczna | ul. Szczytna 20, Torun | 87-100 Poland |
| Jeffrey | Kaine | | Sarasota Arthritis Research Center | 1945 Versailles Street, Suite 101, Sarasota, FL | 34239 USA |
| Danuta | Kapolka | | Slaski Szpital Reumatologiczno-Rehabilitacyjny | ul. Szpitalna 11, Ustron | 43-450 Poland |
| Péter | Keszthely | | Pándy Kalman County Hospital | Semmelweis u. 1., Gyula | H-5700 Hungary |
| Edward | Keystone | | Mount Sinai Hospital, The Rebecca MacDonald Center for Arthritis and Autoimmune Disease | Room #1005, Toronto On, ON | M5G 1X5 Canada |
| Majed | Khraishi | | Nexus Clinical Research | 1 Anderson Ave., St. John's, NF | A1B 3E1 Canada |
| Robert | Kimelheim | | Kimelheim, Robert A | 701 Lawn Avenue, Sellersville, PA | 18960 USA |
| Steven | Kimmel | | West Broward Rheumatology Associates | 7431 North University Drive, Suite 300, Tamarac, FL | 33321 USA |
| Alan | Kivitz | | Altoona Center for Clinical Research | 1125 Old Route 220 N., Duncansville, PA | 16635 USA |
| W Patrick | Knibbe | | Intermountain Research | 600 N. Robbins Rd., Suite 100, Boise, ID | 83702 USA |
| Éva | Koó | | Budai Irgalmasrendi Kórház  II.sz.Rheumatológia | Arpad Fejedelem utja 7, Budapest | H-1023 Hungary |
| Eugeniusz | Kucharz | | Samodzielny Publiczny Szpital Kliniczny nr 7 Slaskiej Akademii Medycznej | ul. Ziolowa 45/47, Katowice | 40-635 Poland |
| Stepan | Kutilek | | CCBR Pardubice | Masarykovo nám. 2667, Pardubice | 530 02 Czech Republic |
| Stephen | Lauter | | RIMA | 3023 North Ballas Road, Suite 500D, St. Louis, MO | 63131 USA |
| Roberto | Leon | | Ponce School of Medicine | 280 Monterrey Street, Ponce | 00716 Puerto Rico |
| Jolanta | Lewandowicz | | Wojewodzki Szpital Specjalistyczny im. M.  Kopernika | ul. Pabianicka 62, Lodz | 93-513 Poland |
| Roger | Lidman | | Center for Arthritis & Rheumatic Diseases PC | 300 Medical Parkway, Suite 112, Chesapeake, VA | 23320 USA |
| H G | Lim | | Scheperziekenhuis | Boermarkeweg 60, Rheumatology, Emmen | 7824 AA Netherlands |
| Jeffrey | Lisse | | University of Arizona, AZ Arthritis Center | 1501 North Campbell Avenue, P.O. Box 245093, Tucson, AZ | 88724 USA |
| Daryl | MacCarter | | Idaho Arthritis and Osteoporosis Center | 520 South Eagle Road, Suite 3211, Meridian, ID | 83642 USA |
| Zelmira | Macejova | | Fakultna nemocnica L.Pasteura 1. interna klinika Kosice | Trieda SNP 1, Kosice | 040 11 Slovakia |
| Michel | Malaise | | Centre Hospitalier Universitaire de Liège | Domaine Universitaire du Sart Tilman, Liège | 4000 Belgium |
| David | Mandel | | David R Mandel, MD, Inc. | 6551 Wilson Mills Road,  Suite 106, Mayfield Village, OH | 44143 USA |
| Richard | Martin | | Arthritis Education and Treatment Center | 1155 East Paris Avenue, Suite 100, Grand Rapids, MI | 49546 USA |
| Emilio | Martín Mola | | Hospital La Paz | Paseo de La Castellana, 261, Madrid | 28046 Spain |
| Steven | Mathews | | Jacksonville Center for Clinical Research | 4085 University Boulevard South, Suite 1, Jacksonville, FL | 32216 USA |
| Marco | Matucci Cerinic | | Ospedale Villa Monnatessa | Viale Pieraccini 18, Firenze | 50100 Italy |
| Tim | McCarthy | | Manitoba Clinic Medical Corporation | 790 Sherbrook Street, Winnipeg, MB | R3A 1M3 Canada |
| Gabriel | Medrano Ramirez | | Clinica de diagnostico y tratamiento de las enfermedades reumaticas SC | Durango #49, Third floor 301, Colonia Roma, Mexico, CP | 06700 Mexico |
| Chandrakant | Mehta | | Mehta, Chandrakant V MD | 949 Calhoun Place, Suite F, Hemet, CA | 92543 USA |
| Zbigniew | Mencel | | SZOZ "Doctor" Sp. z o. o. Poradnia Reumatologiczna | ul. Srodmiejska 34, Kalisz | 62-800 Poland |
| Michael | Miniter | | Quad City Rheumatology SC | 4362 7th Street, Moline, IL | 61265 USA |
| Maria | Misterska-Skora | | NZOZ Materia Medica | ul. Pilsudskiego 23, Wroclaw | 50-044 Poland |
| Wesley | Mizutani | | Talbert Medical Group | 19066 Magnolia Street, Huntington Beach, CA | 92644 USA |
| Peter | Nash | | Rheumatology Research Unit - Coast Joint Care | 9-10 Maroochy Waters Shopping Centre, Denna Street, Maroochydore, QLD | 4558 Australia |
| Federico | Navarro | | Hospital Virgen Macarena | Avenida Dr. Fedriani, s/n., Sevilla | 41009 Spain |
| Jeffrey S | Neal | | Bluegrass Community Research, Inc | 4389 Josiah Way, Lexington, KY | 40515 USA |
| Dariusz | Niedzialek | | Specjalistyczny Osrodek Medycyny Wieku Dojrzalego Sp. z o.o | Al. Dzieci Polskich 20, Warszawa | 04-730 Poland |
| Lea | Pank | | Parnu Hospital Foundation, Clinic of Internal Medicine, Outpatient Department | ul. Ristiku 1, Parnu | 80010 Estonia |
| Raffaele | Pellerito | | Ospedale Mauriziano | Largo Turati 42, Torino | 10100 Italy |
| Aleth | Perdriger | | Centre Hospitalier Universitaire Hôpital Sud | 16 boulevard de Bulgarie,  Rennes | 35200 France |
| Jeffrey | Poiley | | Jeffrey E. Poiley, MD, PA | 324 Park 40 North Blvd., Ste. B, Orlando, FL | 32804 USA |
| Gyula | Poor | | National Institute of Rheumatology | Frankel Leo u. 25-29., Budapest | H-1027 Hungary |
| Janet | Pope | | St. Joseph's Health Care | 268 Grosvenor Street, London, ON | N6R 4V2 Canada |
| Artur | Racewicz | | Niepubliczny Zaklad Opieki Zdrowotnej CENTRUM MEDYCZNE | ul. Pulaskiego 69, Bialystok | 15-337 Poland |
| Frank | Raeman | | Ziekenhuis Netwerk Antwerpen Jan Palfijn | Lange Bremstraat 70, Merksem | 2170 Belgium |
| Maria | Rell-Bakalarska | | Instytut Reumatologii | ul. Spartanska 1, Warszawa | 02-637 Poland |
| Warren | Rizzo | | Advanced Arthritis Care and Research | 10210 North 92nd Street,  Suite 105, Scottsdale, AZ | 85258 USA |
| Janet | Roddy | | Goatcher Clinical Research Unit | Level 2, Thorburn House, Royal Perth Hospital, Shenton Park Campus, Selby Street, Shenton Park, WA | 6008 Australia |
| Jude | Rodrigues | | Clinical Research and Arthritis Centre | 1720 Howard Avenue, Suite 160, Windsor, ON | N8X 5A6 Canada |
| Jose Andres | Roman | | Hospital Doctor Peset | Avenida Gaspar Aguilar, 90., Valencia | 46017 Spain |
| Jan | Rosa | | DC Mediscan | Sustova 1930, Praha 11 | 148 00 Czech Republic |
| Jozef | Rovensky | | Narodny Ustav Reumatickych Chorob | Nabrezie I. Krasku 4, Piestany | 921 12 Slovakia |
| Cristina | Saldate Alonso | | Centro de Investigacion del Noroeste | Ave Guadalupe Victoria 9308-203 B, Tijuana Baja California, CP | 22010 Mexico |
| Carlo | Salvarani | | Arcispedale S. Maria Nuova | Viale Umberto I, 50, Reggio Emilia | 42100 Italy |
| Daina | Saulite-  Kandevica | | D Saulites-Kandevica private practice | ul. Aldaru 20/24, Liepaja, LV | 3400 Latvia |
| Michael | Schiff | | Denver Arthritis Clinic, PC | 200 Spruce Street, Suite 100, Denver, CO | 80230 USA |
| Stuart Norman | Seigel | | Medical Arts Health Research Group | 1605 Gordon Ave, Kelowna, BC | V1Y 3G8 Canada |
| Tom | Sheeran | | Cannock Chase Hospital | Brunswick Road, Staffs | WS11 2XY United  Kingdom |
| William | Shergy | | Rheumatology Associates of North Alabama | 201 Sivley Road, Suite 620,  Huntsville, AL | 35801 USA |
| Jean | Sibilia | | Hopital de Hautepierre | 1 avenue Moliere, Strasbourg Cedex | 67100 France |
| H Arthur | Silverman | | Scripps Clinic | 10666 North Torrey Pines Road, Maildrop MS 113, La Jolla, CA | 92037 USA |
| Suthin | Songcharoen | | Arthritis and Osteoporosis Treatment and Research Center | 2550 Flowood Drive, Suite 300, Flowood, MS | 39232 USA |
| Oscar | Soto-Raices | | San Juan Arthritis and Research Center Incorporated | El Monte Mall-Suite 2010, 652 Ave Munoz Rivera, San Juan | 00918 Puerto Rico |
| Beata | Spengler | | Zala Megyei Kórház | Zrinyi Miklós u. 1, Zalaegerszeg | H-8900 Hungary |
| Andrew | Sulich | | Shores Rheumatology | 29200 Harper Avenue, St. Clair Shores, MI | 48081 USA |
| Gregory | Summers | | Derbyshire Royal Infirmary Rheumatology Dept. | London Road, Derby | DE1 2QY United Kingdom |
| Ferenc | Szanyó | | Petz Aladár County Hospital Department of Rheumatology | Hid u. 2., Györ | H-9025 Hungary |
| Zoltán | Szekanecz | | Debrecen University 3rd Clinic of Internal Medicine, Division of Rheumatology | Moricz Zs. u. 22., Debrecen | H-4004 Hungary |
| Istvan | Szombati | | Synexus Hungary Ltd. Internal medicine | Becsi ut 61, Budapest | H-1036 Hungary |
| James | Taborn | | Midwest Arthritis Center | 1717 Shaffer Street, Suite 124, Kalamazoo, MI | 49048 USA |
| Paul | Tak | | Academisch Medisch Centrum | P O Box 22700, University of Amsterdam F4‑218, Amsterdam | 1100 DE Netherlands |
| Katalin | Takács | | Kiskunhalasi Semmelweis Korhaz Reumatologia | Dr. Monszpart L. u. 1, Kiskunhalas | H-6400 Hungary |
| Suman | Thakker | | Desert Valley Medical Group | 16850 Bear Valley Road  Suite 103, Victorville, CA | 92395 USA |
| Carter | Thorne | | Arthritis Research | 43 Lundy's Lane, Newmarket, ON | L3Y 3R7 Canada |
| Robert | Trapp | | The Arthritis Center - Springfield | 2528 Farragut Drive, Springfiled, IL | 62704 USA |
| Jean Luc | Tremblay | | Centre de Recherche musculo-squelettique de Trois-Rivieres | 1119, Ste-Marguerite, Trois-Rivieres, QC | G8Z 1Y2 Canada |
| Orrin | Troum | | Troum, Orrin M and Medical Associates | 2336 Santa Monica Boulevard, Suite 207, Santa Monica, CA | 90404 USA |
| Filip | Van Den Bosch | | Universitair Ziekenhuis Gent | De Pintelaan 185 K12E, Gent | 9000 Belgium |
| Nathan | Wei | | Arthritis and Osteoporosis Center of Maryland | 71 Thomas Johnson Dr, Frederick, MD | 21702 USA |
| Frank | Wellborne | | Houston Institute for Clinical Research | 7777 Southwest Freeway, #7201, Houston, TX | 77074 USA |
| Lucas | Williame | | Ziekenhuis Netwerk Antwerpen Middelheim | Lindendreef 1, Antwerpen | 2020 Belgium |
